# The Trend of Arterial Carboxyhemoglobin in Non-smokers as a Prognostic Tool in Severe COVID-19 Patients: A Single-Centre Prospective Study

**DOI:** 10.7759/cureus.31955

**Published:** 2022-11-28

**Authors:** Umar H Khan, Amrit Dhar, Suhail Mantoo, Tajamul Shah, Santosh G Rathod

**Affiliations:** 1 Internal and Pulmonary Medicine, Sher-i-Kashmir Institute of Medical Sciences, Srinagar, IND; 2 Clinical Hematology, Sher-i-Kashmir Institute of Medical Sciences, Srinagar, IND

**Keywords:** trend, mortality, arterial blood gas, covid-19, prognosis, carboxyhemoglobin

## Abstract

Introduction

Carboxyhemoglobinemia is characterised by decreased oxygen delivery to tissues. In severe and critical coronavirus disease 2019 (COVID-19) illness with hypoxia, this can herald a grave and protracted course of illness. Patients with COVID-19 experience respiratory impairment, lowering the pace at which carbon monoxide (CO) is eliminated and raising the likelihood of carboxyhemoglobinemia. We set out to explore early arterial carboxyhemoglobin (COHb) and COVID-19 patient outcomes in non-smokers and its potential as a predictive tool for mortality.

Methods

Forty-five patients, non-smokers with severe/critical severe acute respiratory syndrome coronavirus 2 (SARS-CoV-2) infection requiring admission in a North Indian 1200-bedded tertiary care hospital, were recruited prospectively from October 2020 to March 2021. Arterial COHb% was evaluated with arterial blood gases using an analyser, which were taken at the time of admission and then every alternate day for the first 10 days. Carboxyhemoglobinemia was defined as COHb% more than 1%. The primary outcome was defined as the patient’s hospital outcome (survivor/non-survivor).

Results

Of the total 45 subjects, 51.1% (n=23) survived. Patients developed carboxyhemoglobinemia with an incidence of 51% during the course of their hospital stay. The mean ± SD of COHb% on admission was 1.0 ± 0.58 and 1.03 ± 0.8 in non-survivors and survivors, respectively (p=0.870). Maximal individual values of 5.3% and 6.1% were seen in survivors and non-survivors, respectively. On serial COHb measurement, non-survivors had significantly higher COHb% on days 6 and 10. No co-relation of COHb% with inflammatory markers was noted.

Conclusion

Arterial COHb levels in non-survivors were significantly higher than in survivors on days 6 and 10. Our study did not show a prognostic value of serial COHb measurement in patients with severe COVID-19. To establish COHb as a predictive marker in severely ill COVID-19 patients, additional research is required.

## Introduction

Although the majority of coronavirus disease 2019 (COVID-19) patients are asymptomatic or paucisymptomatic, up to 15% of the patients go on to develop a severe or catastrophic illness with pneumonia and hypoxemia. A lower but still sizeable fraction of patients develop acute respiratory distress syndrome (ARDS), which is linked to systemic and/or pulmonary inflammation [1,2].

In addition to daily clinical examinations, individuals with COVID-19 who were hospitalised also require imaging tests such as computed tomography (CT) or chest X-rays to determine the severity or course of the condition. Additionally, a number of biochemical markers have been suggested as indicators of illness severity and development [3]. It has been difficult to find sensitive and precise biomarkers that indicate the severity of the disease and alert professionals to the patient's worsening condition. Having access to essential information on a patient's oxygenation and electrolyte balance in just a few minutes has made arterial blood gases (ABGs) a valuable tool in the management of critically ill patients in the intensive care unit (ICU). This is especially true for COVID-19 inpatients [4]. Therefore, looking into lesser-known ABGs in the context of COVID-19 might help unravel novel prognostic biomarkers.

Heme oxygenase (HO) produces carbon monoxide (CO) endogenously. The inducible form of HO-1 is found in alveolar macrophages, endothelial cells, and epithelial cells. HO-1 is activated by a variety of stimuli, including pro-inflammatory cytokines and nitric oxide (NO) [3-5]. Although CO can be found in measurable amounts in healthy subjects' exhaled air, pulmonary diseases such as bronchial asthma, chronic obstructive pulmonary disease (COPD), bronchiectasis, acute pneumonia, and upper respiratory tract infections increase the levels of inducible HO-1, which produces CO [6-9]. Because arterial CO has a high affinity for haemoglobin (Hb), it can be found as carboxyhemoglobin (COHb). COHb reduces blood oxygen-carrying capacity by shifting the O2 dissociation curve to the left because CO binds to haemoglobin 250 times more avidly. In healthy non-smokers, the typical range is estimated to be between 0.5% and 1.5% [10]. Arterial COHb is found to be co-related with exhaled CO concentration when no exogenous source is identified and has been suggested as a marker in inflammatory pulmonary diseases [6].

Patients with COVID-19 who have carboxyhemoglobinemia may have higher endogenous COHb production or lower endogenous COHb elimination. When breathing is compromised, CO elimination is reduced. Patients with COVID-19 experience respiratory impairment, which lowers the pace at which CO is eliminated and raises the likelihood that carboxyhemoglobinemia may develop [11]. This study explores the connection between early arterial COHb and COVID-19 patient outcomes in non-smokers and explores its potential as a predictive tool.

## Materials and methods

This was a single-centre, prospective, observational study that took place in a 1200-bed tertiary care university hospital, Sher-i-Kashmir Institute of Medical Sciences, Srinagar, Jammu and Kashmir, India, that had dedicated COVID-19 wards and ICUs. Between October 2020 and March 2021, patients were recruited for a six-month period. The Institutional Ethics Committee, Sher-i-Kashmir Institute of Medical Sciences, Srinagar, India, approved the study (approval number: #RP/09/2020) and all procedures followed the Helsinki Declaration. After receiving written informed consent, the participants were enrolled. 

**Figure 1:**
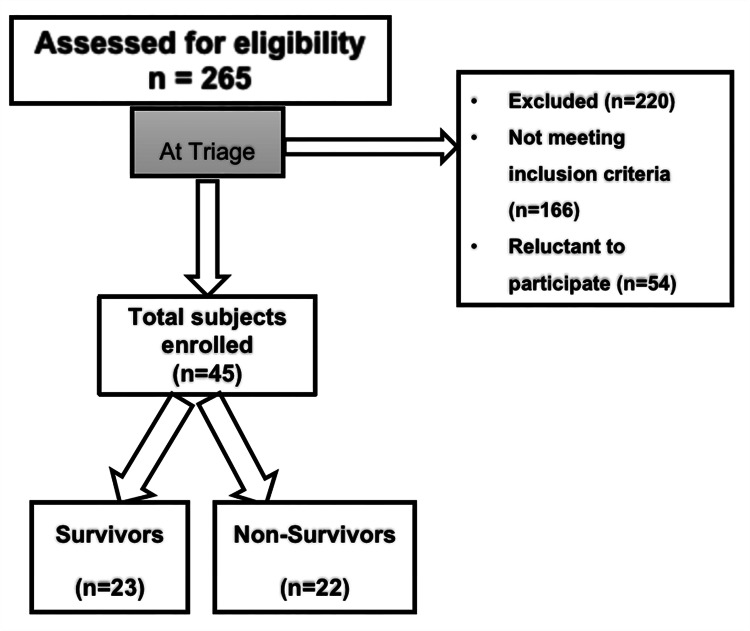
STROBE flow diagram depicting patient enrolment. STROBE: Strengthening the Reporting of Observational Studies in Epidemiology

A total of 265 COVID-19 patients were screened for eligibility for participation in this study. Forty-five patients who met the inclusion criteria were eventually enrolled. Inclusion criteria included age more than or equal to 18 years, non-smokers (including ex-smokers who quit more than one year ago), SARS-CoV-2 positive by nasopharyngeal swab test, severe and/or critical COVID-19 [12], and those with less than 24 hours of admission to the hospital. Specific treatment for COVID-19 varied according to international recommendations and local institutional protocols. Carboxyhemoglobenemia was defined as COHb% more than 1%. Severe illness was defined as individuals who have oxygen saturation (SpO2) <94% on room air at sea level, a ratio of arterial partial pressure of oxygen to fraction of inspired oxygen (PaO2/FiO2) <300 mm Hg, respiratory frequency >30 breaths/minute, or lung infiltrates >50%. Critical illness is defined as individuals who have respiratory failure, septic shock, and/or multiple organ dysfunction [13]. Figure 1 depicts the STROBE (Strengthening the Reporting of Observational Studies in Epidemiology) flow diagram depicting the enrolment of patients in the study.

Data collection

Data on demographics, underlying co-morbidities, vital signs, the clinical severity of COVID-19 on admission, WHO grade of severity on admission [13], ICU severity scores (Sequential Organ Failure Assessment (SOFA) and Acute Physiology and Chronic Health Evaluation II (APACHE-II)), laboratory investigations including inflammatory markers (C-reactive protein, ferritin, D-dimer, IL-6), medical treatment, and oxygen delivery system used for the patient at the time of admission were collected by the investigators.

Arterial COHb was evaluated using ABG, which were taken at the time of admission and then every alternate day for the first 10 days (day 2,4,6,8,10), with day 0 being the day of admission. The primary outcome was defined as the patient's mortality (survivor/non-survivor). For patients who died before day 10, the last CoHb analysis was on the day of death. ABG analysis was done using the Siemens® RapidLab 1245 analyser (Siemens Healthineers AG, Erlangen, Germany). Calibration was performed daily.

Statistical analysis

For descriptive statistics, the continuous variable was summarised as mean standard deviation (SD) or median and interquartile range (IQR) as appropriate. The categorical variables are described as frequencies and percentages. All data were assessed for normality. Student’s t-test, Fisher's exact test, and Mann-Whitney U test were used wherever applicable. Spearman’s correlation was used for ordinal variables, while Pearson’s correlation was used for continuous variables. Mixed method ANOVA with repeated measures was used for trend analysis between the two groups. Statistical analysis was done using IBM SPSS Statistics for Windows, Version 28.0 (Released 2021; IBM Corp., Armonk, New York, United States). The significance level was set at p < 0.05.

## Results

Of the total 45 subjects, 51.1% (n=23) survived. Table 1 represents the baseline characteristics of subjects, with a comparable median age between the non-survivors and survivors (p=0.768). The mean ± SD of COHb% on admission was 1.0 ± 0.58 and 1.03 ± 0.8 in non-survivors and survivors, respectively (p=0.870). Maximal individual values of 5.3% and 6.1% were seen in survivors and non-survivors, respectively. During the course of their hospital stay, patients developed carboxyhemoglobinemia with an incidence of 51%. The SOFA and APACHE II scores and WHO grade in non-survivors and survivors were not significant. There were no significant differences in the neutrophil-to-lymphocyte ratio (NLR), D-dimer, and lactate dehydrogenase (LDH) levels in the two groups. Interleukin 6 (IL-6) yielded a statistically significant difference between survivor and non-survivor groups (p=0.043). 

**Table 1:** Baseline characteristics of non-survivors and survivors IQR: interquartile range; SD: standard deviation; COPD: chronic obstructive pulmonary disease, SOFA: sequential organ failure assessment; APACHE-II: acute physiological and chronic health evaluation; CTSI: computed tomography severity index; MV: mechanical ventilation; HFNO: high flow nasal oxygenation; IL-6: interleukin 6; LDH: lactate dehydrogenase; NLR: neutrophil-to-lymphocyte ratio For continuous variables, student's t-test and Mann-Whitney U test were used; for categorical variables, Fischer's exact test and chi-square test were used.

Patient characteristics	Non-survivors (n=22)	Survivors (n=23)	p value
Age, median (IQR), years	70 (60.75-75)	60 (60-70)	0.768
Males, (n %)	12 (54.5)	12 (52.2)	0.873
BMI (kg/m^2^), mean ± SD	28.57 ± 3.87	28.32 ± 4.18	0.650
Hypertension, (n %)	16 (72)	14 (63)	0.530
Diabetes mellitus, (n %)	10 (45)	8 (36)	0.335
COPD, (n %)	3 (13)	6 (26)	0.252
WHO grade on admission, median (IQR)	4 (3-4)	3 (3-4)	0.339
COHb% on admission, mean ± SD	1.0 (0.58)	1.03 (0.8)	0.870
SOFA, median (IQR)	3.5 (3-6.5)	3 (2-4)	0.121
APACHE-II, median (IQR)	9 (6.75-14.25)	8 (5-10)	0.065
CTSI, median (IQR)	13 (13-18)	12 (10-13)	0.001
Need for MV/HFNO, (n %)	22 (100%)	7 (30%)	<0.001
NLR (IQR))	9.3 (5-23)	8.3 (5.2-22.8)	0.892
IL-6 pg/ml, median (IQR)	37.21 (11.23-80.16)	17.21 (8.35-31.46)	0.043
D-dimer ng/dl, median (IQR)	640 (362.25-1266)	399 (270-1008)	0.238
LDH IU/L, median (IQR)	463.5 (296.25-620.75)	318 (257-480)	0.140

**Figure 2:**
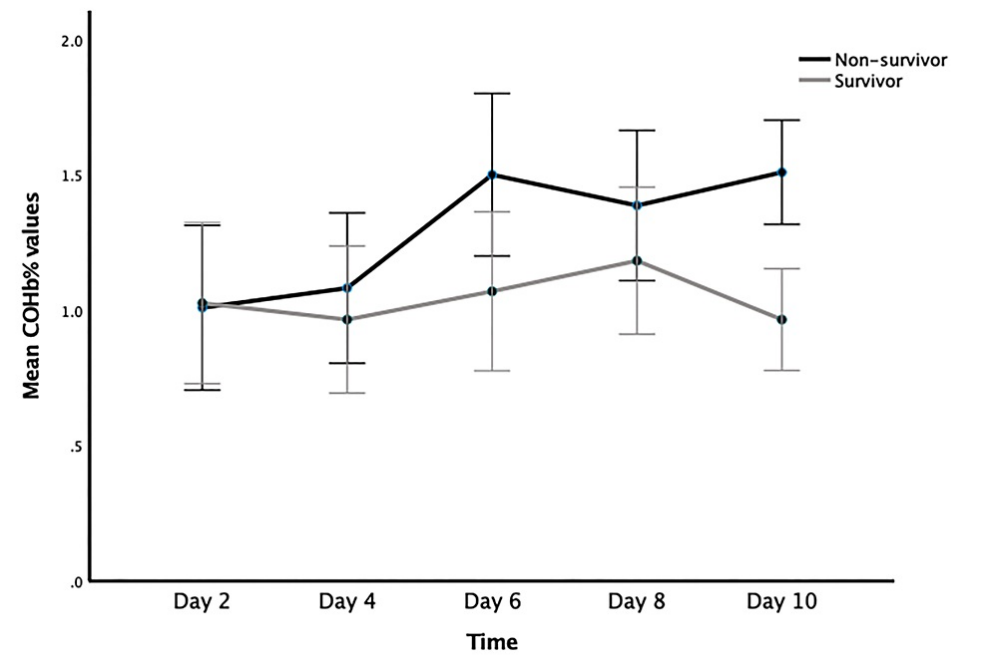
Trend analysis of mean COHb% between non-survivors and survivors over 10 days of admission. Error bars represent 95%CI. Significance was noted on day 6 and day 10 ( p< 0.05) COHb: carboxyhemoglobin; CI: confidence interval

Table 2 depicts mean ± SE (standard error) of COHb% at day 2,4,6,8,10, along with 95% confidence intervals (CI) between the two groups. Non-survivors had a significantly higher COHB% on day 6 and day 10 compared to survivors (p<0.05) as depicted in Figure 2. 

**Table 2:** Mean COHb% at alternate days of admission between non-survivors and survivors. Significance was noted on days 6 and 10. SE: standard error; CI: confidence interval; COHb: carboxyhemoglobin

Day of admission	Non-survivor (n=22)		Survivor (n=23)		p-value
	Mean ± SE	95% CI	Mean ± SE	95% CI	
Day 2	1.009 ± 0.151	0.705, 1.313	1.026 ± 0.147	0.729, 1.323	0.936
Day 4	1.082 ± 0.138	0.804, 1.359	0.965 ± 0.135	0.694, 1.237	0.548
Day 6	1.500 ± 0.149	1.200, 1.800	1.070 ± 0.146	0.776, 1.363	0.045
Day 8	1.386 ± 0.137	1.109, 1.664	1.183 ± 0.134	0.911, 1.454	0.295
Day 10	1.509 ± 0.095	1.317, 1.701	0.965 ± 0.093	0.777, 1.153	<0.001

**Figure 3:**
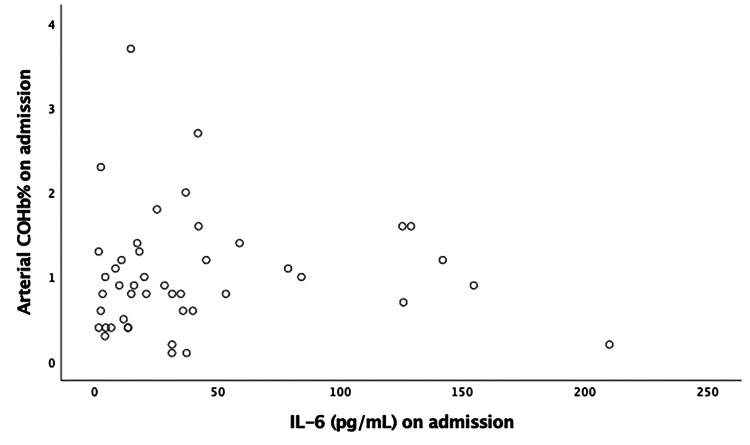
Pearson’s correlation of carboxyhemoglobin on admission with IL-6 (r = -0.005, p= 0.972). COHb: carboxyhemoglobin; IL-6: interleukin 6

**Figure 4:**
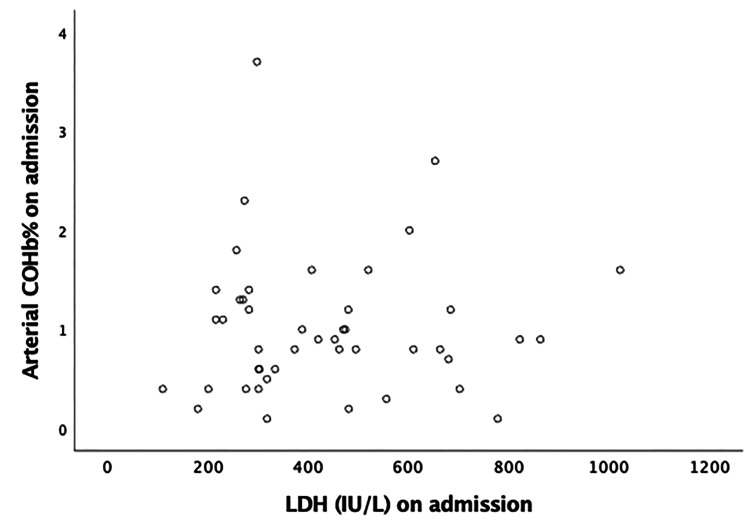
Pearson’s correlation of carboxyhemoglobin on admission with LDH (r = 0.025, p= 0.870). COHb: carboxyhemoglobin; LDH: lactate dehydrogenase

**Figure 5:**
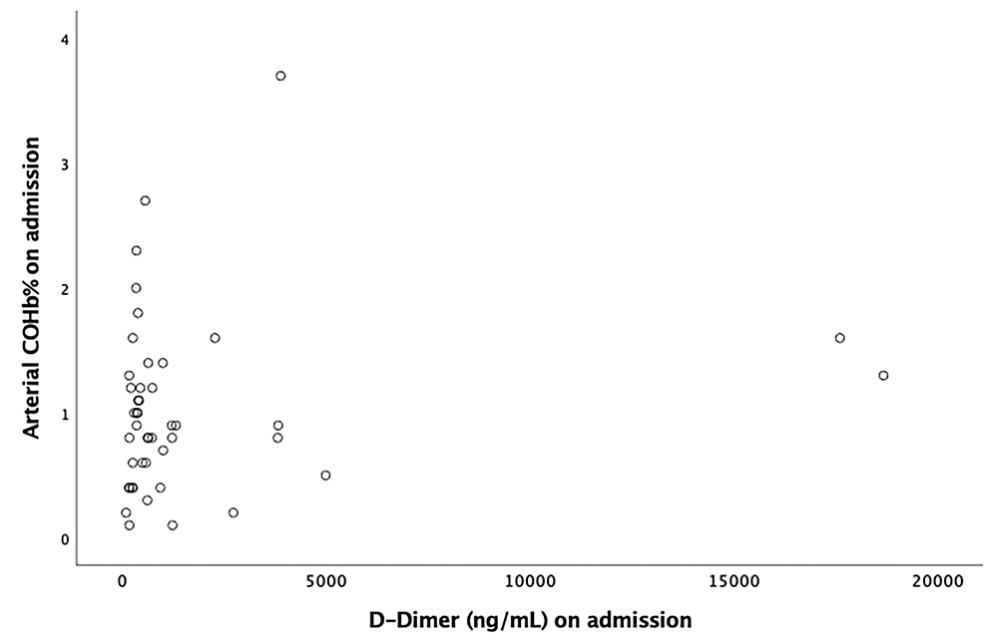
Pearson’s correlation of carboxyhemoglobin on admission with D-dimer (r = 0.168, p= 0.269). COHb: carboxyhemoglobin

**Figure 6:**
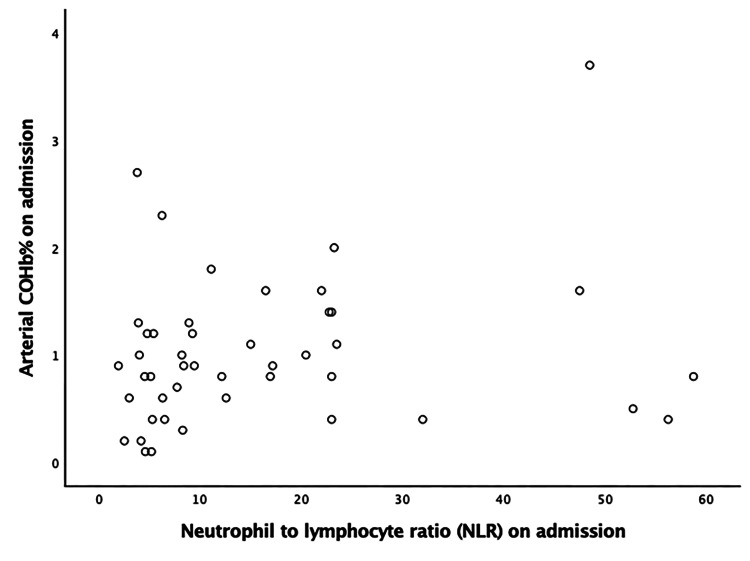
Pearson’s correlation of carboxyhemoglobin on admission with NLR (r =0.187, p= 0.219). NLR: neutrophil-to-lymphocyte ratio; COHb: carboxyhemoglobin

The correlation of COHB% on admission with IL-6, LDH, D-dimer, and NLR is depicted in Figures 3-6, respectively. No significant correlation was found. The relationship between arterial COHb with inflammatory markers (IL-6, D-dimer, LDH, NLR), SOFA and APACHE II scores, and WHO grade was investigated at different time points (on admission, day 5, and day 10), and no correlation was found. 

## Discussion

Carboxyhemoglobinemia is a serious condition characterised by decreased oxygen delivery to tissues. It can exacerbate the course of COVID-19 in hypoxic patients. Due to the scarcity of data on carboxyhemoglobinemia in COVID-19 and its pathogenesis, we wanted to see if arterial carboxyhemoglobin levels could be used as a prognostic marker in severe/critical COVID-19.

In our study, carboxyhemoglobinemia was defined as arterial COHb of more than 1%, with an incidence of around 51% during the hospital stay. According to Faisal et al., the incidence of carboxyhemoglobinemia in critically ill COVID-19 patients was around 26.7%; carboxyhemoglobinemia was defined as COHb>2% in nonsmokers and COHb>5% in smokers [14]. The difference in COHb cutoff values in critically ill COVID-19 patients could explain why carboxyhemoglobinemia was more common in our study. Furthermore, providing these patients with oxygen supplementation and/or mechanical ventilation increases the rate of CO elimination and calls into question the outpatient or ambulatory definitions of carboxyhemoglobinemia. Nonetheless, the disparity between studies necessitates agreement on the definition of carboxyhemoglobinemia in the context of COVID-19.

Our findings that COHb levels were greater in non-survivors on days 6 and 10 were confirmed by a related study by Ledoux et al., which reported statistically higher values in non-survivors than survivors throughout the hospital stay [15]. One of the main causes of death in COVID-19 patients is cytokine release syndrome (CRS) is frequent in patients with severe/critical illnesses, which has been observed during the second week of illness [16,17]. The rise in COHb% shown in non-survivors around day 6 in our study could be explained by these cytokines, an oxidative stress-mediated pro-inflammatory state, high HO-1 expression, and these factors combined. Additionally, non-survivors in the second week of illness displayed a decrease in oxygenation and impaired respiratory ability to eliminate COHb due to the development of ARDS, underlying sepsis [18-20], and hemolysis (drug and viral-illness mediated). As a result, higher values were noted around day 6 and 10, in comparison to survivors who began showing improvement in oxygenation, and overall SOFA and APACHE II scores, which were positively correlated, but not statistically significant. 

A higher-than-optimal level of COHb may reduce mortality in severely ill medical and surgical patients, according to a number of researchers from the past [21]. Patients who survived a brief ICU stay after cardiothoracic surgery had a minimum COHb level that was considerably greater than non-survivors, according to a study by Melly et al. [22]. This was contrary to our study, which found a marginally elevated level in critically ill COVID-19 non-survivors. This, too, can be attributed to underlying hemolysis [23], oxidative stress, sepsis, and the subsequent upregulation of heme oxygenase in the liver, spleen, and lungs, which together cause an increase in endogenous CO production [18-20].

Changes in the ventilator settings and the inspired oxygen fraction must also be taken into consideration as they may have an impact on arterial COHb concentration [24]. The struggle between CO and O2 for the identical binding sites will naturally cause a temporary increase in lung removal of CO when the inspired oxygen percentage rises. Since the oxygen needs increased during the acute first stages of the illness, this should have led to higher COHb clearance and lower arterial levels. However, a number of factors influence how the CO produced endogenously is eliminated. These include endogenous CO generation, CO catabolism, alveolar ventilation, lung capillary oxygen pressure, and CO lung diffusing capacity. It remains debatable whether the increase in COHb can be attributed to increased production or impaired CO elimination via the alveoli-capillary membrane. 

Overall, there are many causes for COHb levels to increase in a COVID-19 patient. Despite the fact that our study showed that non-survivors had greater levels than survivors, we still do not advise utilising COHb levels as a prognostic indicator in severe COVID-19 sickness. There appears to be no link between COHb levels and COVID-19 pneumonia outcomes in terms of mortality, need for mechanical ventilation, or hospitalisation, according to other studies done on the same topic [25,26].

Our study has some limitations. These include the small cohort size and the fact that only day 10 of hospitalization's worth of serial measures were taken. Additionally, it's possible that treatments like mechanical ventilation and oxygen supplementation helped the lungs eliminate more CO. It would be immoral to withhold oxygen treatment from severely ill patients for the sake of the study, even though this might have had an impact on the quality of the data gathered. Future research should concentrate on defining carboxyhemoglobinemia in COVID-19 patients on a consistent basis and examining the relationship between it and disease progression over a longer time horizon and in a bigger patient cohort. Further research should be done to determine how anti-inflammatory medications that target the conditions that cause carboxyhemoglobinemia (cytokine storm, ARDS, sepsis, etc.) affect COHb levels. 

## Conclusions

COHb levels in non-survivors were significantly higher than in survivors on days 6 and 10. There was no association between inflammatory markers and arterial COHb%. Our study did not demonstrate a prognostic value for serial COHb measurement. In order to establish COHb as a predictive marker in severely ill COVID-19 patients, further research is required.
